# E-Cadherin-Deficient Cells Are Sensitive to the Multikinase Inhibitor Dasatinib

**DOI:** 10.3390/cancers14071609

**Published:** 2022-03-22

**Authors:** Nicola Bougen-Zhukov, Lyvianne Decourtye-Espiard, Wilson Mitchell, Kieran Redpath, Jacqui Perkinson, Tanis Godwin, Michael A. Black, Parry Guilford

**Affiliations:** Centre for Translational Cancer Research (Te Aho Matatū), Cancer Genetics Laboratory, Department of Biochemistry, University of Otago, Dunedin 9016, New Zealand; nicola.bougen-zhukov@otago.ac.nz (N.B.-Z.); lyvianne.decourtye@otago.ac.nz (L.D.-E.); mitwi509@student.otago.ac.nz (W.M.); redki406@student.otago.ac.nz (K.R.); jacquiperkinson96@gmail.com (J.P.); tanis.godwin@otago.ac.nz (T.G.); mik.black@otago.ac.nz (M.A.B.)

**Keywords:** HDGC, E-cadherin, lobular breast cancer, diffuse gastric cancer, synthetic lethality, chemoprevention, dasatinib, AKT serine/threonine kinase 3 AKT3, discoidin domain receptor 2 (DDR2)

## Abstract

**Simple Summary:**

Inactivating mutations in the *CDH1* gene, encoding the cell adhesion protein E-cadherin, cause hereditary diffuse gastric cancer syndrome (HDGC), as well as being a hallmark of sporadic diffuse gastric cancers and lobular breast cancers. We have previously identified *AKT3* as a potential vulnerability in gastric cancers lacking *CDH1* expression. This study aimed to test whether drugs which inhibit the *AKT3*-associated gene, discoidin domain receptor tyrosine kinase 2 (*DDR2*) specifically targeted cells deficient in E-cadherin. We demonstrated that cells and organoids lacking E-cadherin exhibited heightened sensitivity to dasatinib, a drug that targets multiple kinases including DDR2 and SRC.

**Abstract:**

The *CDH1* gene, encoding the cell adhesion protein E-cadherin, is one of the most frequently mutated genes in gastric cancer and inactivating germline *CDH1* mutations are responsible for the cancer syndrome hereditary diffuse gastric cancer (HDGC). *CDH1*-deficient gastric cancers exhibit high AKT serine/threonine kinase 3 (*AKT3)* expression, but specific drugs against this AKT isoform are not available. We therefore used two publicly available datasets to identify *AKT3*-associated genes which could be used to indirectly target AKT3. Reactome analysis identified an enrichment of extracellular matrix remodelling genes in *AKT3*-high gastric cancers. Of the 51 genes that were significantly correlated with *AKT3* (but not *AKT1*), discoidin domain receptor tyrosine kinase 2 (*DDR2*) showed the strongest positive association. Treatment of isogenic human cells and mouse gastric and mammary organoids with dasatinib, a small molecule inhibitor of multiple kinases including SRC, BCR-ABL and DDR2, preferentially slowed the growth and induced apoptosis of E-cadherin-deficient cells. Dasatinib treatment also preferentially slowed the growth of gastric and mammary organoids harbouring both *Cdh1* and *Tp53* mutations. In organoid models, dasatinib treatment was associated with decreased phosphorylation of total AKT, with a stronger effect seen in *Cdh1*-deficient organoids. Treatment with combinations of dasatinib and an inhibitor of AKT, MK2206, enhanced the effect of dasatinib in breast MCF10A cells. In conclusion, targeting the DDR2-SRC-AKT3 axis with dasatinib represents a promising approach for the chemoprevention and chemotherapy of gastric and breast cancers lacking E-cadherin.

## 1. Introduction

Germline mutations in *CDH1*, the gene encoding the cell adhesion protein E-cadherin, are responsible for the cancer syndrome hereditary diffuse gastric cancer (HDGC) [1]. HDGC was first described in three extended New Zealand Māori families in 1998 [2]. The lifetime risk of diffuse gastric cancer (DGC) in HDGC is estimated to be approximately 56% for women and 70% for men [3], although there is considerable variation reported between families; estimates from clinical genetic laboratories based on multi-gene panel testing range from 25–33% for women and 37–42% for men [4,5] whereas lifetime risks of 67% and 83% for men and women, respectively, were reported in one early series of stand-out families [6]. In addition to DGC, female germline *CDH1* mutation carriers have a lifetime risk of lobular breast cancer (LBC) estimated at 39–55% [3,4,5,6]. Additionally, somatic *CDH1* mutations are present in approximately 50% of sporadic cases of DGC [7], and loss of E-cadherin is a hallmark of lobular carcinoma in situ (LCIS) and LBC [8]. Approximately 90% of LBCs lack E-cadherin protein expression [9].

It is recommended that pathogenic *CDH1* mutation carriers from HDGC families undergo a prophylactic gastrectomy [1]. However, this procedure can result in long term morbidity with common complications including lactose intolerance, fat malabsorption, nutritional deficiencies, weight loss and dysphagia [1]. Annual gastroscopic surveillance is recommended for mutation carriers declining or deferring surgery. Unfortunately, there are no chemoprevention methods that can be used to further reduce the risk of advanced gastric cancer in these families, with the possible exception of *H. pylori* eradication [10]. Annual magnetic resonance imaging (MRI) and mammographic surveillance are recommended for the management of the breast cancer risk in HDGC families, although bilateral, risk-reducing mastectomy can be considered [1]. The only current option for chemoprevention of LCIS/LBC in the context of HDGC is treatment with selective oestrogen receptor modulators (SERMS) or aromatase inhibitors. Although there is no direct data on HDGC-associated breast cancers, these anti-oestrogen drugs reduce risk by approximately 50% in women at elevated risk of developing LBC [1,11]. Unfortunately, chemopreventative endocrine therapy can have a significant impact on quality of life, particularly when continued over many years [12]. Moreover, the uptake of these drugs may be compromised in mutation carriers who have undergone a prophylactic gastrectomy, requiring serum drug concentration monitoring and, if required, dose modifications to ensure efficacy is maintained [13]. Genotype analysis for the drug metabolising enzyme CYP2D6 may also be useful for patients receiving tamoxifen [14].

Sporadic DGC and LBC frequently present at an advanced stage, and treatment options are therefore often limited. The standard of care for sporadic, locally advanced gastric cancer usually involves perioperative chemotherapy with docetaxel, oxaliplatin, fluorouracil and leucovorin [15]. However, there is growing evidence that patients with the diffuse subtype receive little benefit from this or other standard chemotherapies [16,17,18]. Traditional chemotherapeutic drugs are also of limited benefit to LBC, as the majority of these tumours have low proliferation rates [19] as well as relatively low mutational burden and the diffuse nature of these tumours also provides challenges for surgical resection [20]. Around 90% of LBCs are oestrogen receptor-positive and frequently respond well initially to adjuvant endocrine therapy with SERMS or aromatase inhibitors, however, a subset of LBC patients do not benefit from adjuvant endocrine therapy [21]. Resistance to the SERM tamoxifen can develop due to a number of factors including expression of oestrogen receptor-gamma as well as enhanced growth factor signalling [22]. Overall, there is a clear need for new chemoprevention and chemotherapy options for both hereditary and sporadic DGCs and LBCs lacking E-cadherin expression.

We have been taking a synthetic lethal approach to specifically target cells lacking E-cadherin expression [23,24] including a recent study that identified a synthetic lethal relationship between allosteric pan AKT inhibitors and *CDH1* expression in breast and gastric cell lines and mouse gastric organoids [25]. Gene expression analysis of gastric tumour samples from The Cancer Genome Atlas (TCGA) stomach adenocarcinoma (STAD) cohort revealed that the expression of the *AKT3* isoform (but not *AKT1* or *AKT2*) is inversely correlated to the expression of *CDH1* in gastric tumours [25]. This suggested that the sensitivity of *CDH1*-null cells to the pan AKT inhibitors may be explained more by the inhibition of AKT3 rather than AKT1 and AKT2, and therefore that over-expression of AKT3 represents a vulnerability in tumours deficient in E-cadherin. AKT3 is over-expressed in a number of other solid tumours, including breast cancers [26,27], and it has been shown to promote cell growth and an epithelial–mesenchymal transition in colorectal and gastric cancer cell lines [28,29]. In addition, shRNA knockdown of *AKT3* inhibited triple negative breast cancer cell spheroid growth and tumour formation in in vivo xenograft studies [30].

Since there are no isoform-specific small molecule inhibitors of AKT3, we sought to identify an indirect route to target this protein in E-cadherin-deficient cells by identifying *AKT3*-associated genes in *CDH1*-low gastric tumours. This analysis identified the discoidin domain receptor 2 (*DDR2*) as an *AKT3*-associated gene. DDRs are unique receptor tyrosine kinases that are activated by binding of collagen components of the extracellular matrix (ECM), instead of soluble peptide growth factors [31]. Here, we examined the sensitivity of E-cadherin-deficient cells to multikinase inhibitors which target DDR2 and identified dasatinib treatment as having a significantly synthetic lethal effect in our cell and organoid models. Therefore, we postulate that the indirect inhibition of AKT3 via DDR2 could be a novel chemopreventative and chemotherapeutic approach for the treatment of E-cadherin-deficient breast and gastric cancers.

## 2. Materials and Methods

### 2.1. Bioinformatics

#### 2.1.1. Datasets and Processing

The Cancer Genome Atlas (TCGA) Stomach Adenocarcinoma (STAD) cohort [32], and the Cristescu cohort (“GEO”) [33] from the Gene Expression Omnibus (GEO—accession GSE652254) were used for differential expression (DE) analysis. Gene level count data for the TCGA cohort were downloaded from the TCGA data portal and processed using the “voom” methodology from the limma (linear models for microarray analysis) package [34] within the statistical computing environment R, version 4.0.4 [35]. Raw microarray data from the Cristescu cohort were downloaded from the NCBI GEO data portal, and were normalised using the robust multiarray analysis (RMA) [36] approach.

#### 2.1.2. Differential Expression Analysis

Differential expression analysis was carried out using the limma package for R, in order to identify differences in gene expression between samples with moderate-to-high (top 66.6%) vs. low (bottom 33.3%) expression of AKT3 within the GEO and TCGA datasets. The Benjamini–Hochberg procedure was employed to provide false discovery rate control, and an adjusted *p*-value of 0.05 was used to define significant differential expression. Genes achieving significance were further filtered by requiring an expression fold change (FC) of at least 2-fold up- or down-regulation. These analyses were repeated for AKT1, but without filtering for expression FC. Once a list of differentially expressed genes had been generated for each of AKT1 and AKT3, any ATK1-associated genes were removed from the AKT3-associated gene list, to avoid selecting compounds that target both CDH1+ and CDH1− gastric isoforms, as these were considered unlikely to be suitable for a targeted therapy approach. The top-ranked gene across both data sets (DDR2) was then selected as a potential target for further analysis.

#### 2.1.3. Reactome Analysis

Lists of significantly differentially expressed genes (FDR adjust *p*-value < 0.05) were used for pathway enrichment analysis, in order to identify Reactome [37] pathways that were overrepresented among genes associated with *AKT3*. The methodology from the goseq package [38] for R was utilised to account for the gene-length bias that is known to impact enrichment analyses in RNA-seq data sets.

### 2.2. Cell Culture

MCF10A cells (CRL 10317), a non-tumorigenic mammary epithelial cell line, and the derived isogenic line with *CDH1* knockout (MCF10A-*CDH1*^−/−^) (#CLLS1042) were purchased from Sigma Aldrich (St. Louis, MO, USA). The MCF10A isogenic lines were cultured in DMEM/F12: (1:1) (Invitrogen, Carlsbad, CA, USA) with 5% horse serum (Invitrogen), 10 μg/mL Actrapid neutral insulin (Novo Nordisk Pharmaceuticals Ltd. Gatwick, West Sussex, UK), 20 ng/mL human epidermal growth factor (Peprotech, Rehovot, Israel), 100 ng/mL cholera toxin and 500 ng/mL hydrocortisone (Sigma Aldrich, St. Louis, MO, USA).

### 2.3. Small Molecule Inhibitors and Chemicals

The kinase inhibitors dasatinib (#AOB87355), imatinib mesylate (#AOB6752), nilotinib (#AOB87155) and ponatinib (#AOB87302), were purchased from AOBious (Gloucester, MA, USA) and the powders reconstituted in sterile DMSO, aliquoted and stored at −20 °C until use. Endoxifen (E8285) was purchased from Sigma. Information regarding the components utilised for organoid media are detailed in Table S1.

### 2.4. Nuclei Enumeration Assay

MCF10A-WT/MCF10A-*CDH1*^−/−^ and NCI-N87/NCI-N87-*CDH1*^−/−^ cells were seeded in 96-well black plates (Greiner, Kremsmünster, Austria) at 4 (MCF10A) or 10 (NCI-N87) × 10^4^ cells per well and left to attach overnight. The next day, outer wells of plates were stained with 1 µg/mL Hoechst 33,342 in PBS for 30 min and imaged on a Cytation 5 imager (Biotek, Winooski, VT, USA). Nuclei were counted at this stage to ensure plating accuracy. If the ratio of WT: *CDH1*^−/−^ cells was >0.65 and <1.3, then plates proceeded to drugging. Cells were drugged with a dose response of compounds as previously described [39] and incubated for a further 48 h, cells were then fixed and stained with 1 µg/mL Hoechst 33,342, 0.25% paraformaldehyde and 0.075% saponin in PBS. Six fields/well at 4× magnification were captured using the Cytation 5 imager (Biotek). Nuclei were counted using Gen5 software (Biotek), and normalised to the vehicle control for each cell line.

MCF10A-WT/MCF10A-*CDH1*^−/−^ cells were assessed for the efficacy of synergistic combinations utilising identical methodology to the nuclei enumeration assay, with the following modifications. Cells were seeded at 1 × 10^3^ cells per well in 384-well, black-walled, clear-bottom tissue culture plates (Greiner Bio-One, Frickenhausen, Germany) in 30 µL of complete growth medium. MCF10A WT and *CDH1*^−/−^ cells were drugged with 10µL of a dose response of compounds; drug 1 + drug 2 vehicle control, drug 2 + drug 1 vehicle control and drug 1 + 2 in combination, as previously described [23] and incubated for a further 48 h. Combination index (CI) values were calculated using CompuSyn software. All experiments were performed as biological triplicates for combination treatments, with replicates of single agents accrued to provide standardised 9-point MCF10A WT and *CDH1*^−/−^ viability measurements, allowing for accurate determination of combination synergism. Single agent standardised viability measurements comprised of 16 replicates minimum, with at least two measurements included from the same plates as the combination biological triplicates these single agents encompassed.

### 2.5. FACS

MCF10A-WT and MCF10A-*CDH1*^−/−^ cells were seeded at 4 and 5 × 10^4^ cells per well, respectively, in 6-well plates. The next day, cells were drugged with dasatinib and left for 48 h. The day before analysis, control wells were drugged with Staurosporine (0.1 µM). On the day of analysis, cells were trypsinised, spun down at 1500 rpm for 5 min, and resuspended in 500 µL Annexin-binding buffer (10 mM Hepes (pH 7.4), 140 mM NaCl, 2.5 mM CaCl_2_). Cells were stained with propidium iodide (Sigma Aldrich, St. Louis, MO, USA) and FITC-Annexin-V (#556420, BD Biosciences, San Jose, CA, USA) for 15 min in the dark. Samples were analysed on BD Fortessa Flow cytometer (BD Biosciences, Franklin Lakes, NJ, USA). Compensation controls were: Untreated unstained, heat-treated cells stained with PI, and Staurosporine (0.1 µM)-treated cells stained with FITC-Annexin-V. Data analysis was performed on FlowJo software v10 (BD Biosciences).

### 2.6. Organoid Culture

Organoids were generated using the stomachs and mammary glands of 6–8 week old mice and an inducible Cre-lox system [40] controlling both *Cdh1* deletion and the activation of the fluorescent marker protein TdTomato under the CD44 stem cell promotor; CD44-Cre/*Cdh1*^(fl/fl)^/tdTomato^(fl/fl)^. Single loxP sites flank a region encompassing exons 6 through 10 of *Cdh1* followed by a floxed selection cassette which is excised via in vitro Cre mediated recombination. These organoids are referred to as *Cdh1*^−/−^ in this manuscript. Control organoids derived from CD44-Cre/tdTomato mice are referred to as WT. An additional mouse strain CD44-Cre/*Cdh1*^(fl/fl)^/*Tp53* ^(fl/fl)^/tdTomato^(fl/fl)^ with inducible simultaneous knockout of *Tp53* and *Cdh1* was also generated in order to obtain organoids deficient in both Tp53 and E-cadherin (organoids derived from these mice referred to as *Tp53*^−/−^*Cdh1*^−/−^ in this manuscript).

#### 2.6.1. Mammary Organoid Generation

To generate mammary organoids form our mouse models, we adapted a protocol from Ewlad et al. [41]. For a detailed protocol refer to Luxenburger and Bougen-Zhukov et al., 2021 [42]. Mammary glands were harvested from virgin female mice and pooled in sterile PBS (+ primocin, (Invivogen, San Diego, CA, USA)) on ice, minced and then incubated (at 100 rpm, 37 °C) with a digestion solution (5% FBS, 2.5 µg/mL Gentamycin, 5 µg/mL insulin, 3 mg/mL dispase II, 1.5 mg/mL collagenase dissolved in Advanced DMEM/F12 media) for 30 min. Tissues were then resuspended in DMEM/F12 (+primocin), centrifuged and resuspended in 4 mL DNase solution (4 U/mL DNase I in DMEM/F12). Tubes were shaken vigorously by hand for 2 min, then 6 mL DMEM/F12 (+primocin) added per tube. Following this, samples were washed four times with DMEM/F12 (+primocin) to remove enzymes and to separate unwanted single cells from organoids. After a final spin at the supernatant was removed, and the pellet was resuspended in 80 µL of matrigel and seeded in 24 well plate.

#### 2.6.2. Gastric Organoid Generation

Gastric organoids were generated using the same procedure as in Brew et al. [23]. Briefly, stomachs were harvested from mice, washed with PBS containing primocin and the keratinised region was removed. The remaining tissue was incubated in chelating buffer (25 mM EDTA, pH 8.0 to loosen glands) at 4 °C for 60–90 min with agitation. The glands were then separated out with vigorous shaking in fresh cold PBS. Large pieces of tissue were removed and glands were spun down at 200× *g* for 5 min at 4 °C. The supernatant was removed and the pelleted glands resuspended in 50 µL matrigel and plated into 24 well plates.

Once the matrigel was set, 500 µL of organoid media was added (for tissue specific formulations please see Table S1 in Supplementary materials) and plates incubated at 37 °C, 5% CO_2_. After glands had started to expand, they were passaged via incubation with 0.05% trypsin (gastric organoids; 10 min, 37 °C) or 0.25% trypsin (mammary organoids; 15 min, 37 °C) to create single cells, counted and seeded in 24 well plates at 1000 cells/well (gastric) and 3000 cells/well (mammary). For routine maintenance, gastric organoids were passaged every 6 days and mammary organoids every 7–9 days.

### 2.7. Drug Treatment of Organoids

Single cell suspensions were generated from gastric and mammary organoids cultures (as described above). After counting, cells were suspended in matrigel and cells were plated in 12.5 µL matrigel per well of 384 well plates at 200 (gastric) and 300 (mammary) cells per well. Once the matrigel was set (approximately 10 min), 20 µL of complete organoid media (tissue specific) was added per well. The expression of TdTom and/or knockout of *Cdh1* (or *Tp53*/*Cdh1*) was induced with endoxifen by addition of 20 µL of 1 µM endoxifen/well-final concentration 0.5 µM the day after seeding (gastric) or three days after seeding (mammary). Then, 24 h (gastric) or 48 h (mammary) after induction, organoids were drugged with dasatinib by addition of 40 µL of 2× concentrated dasatinib with a final concentration of 5–20 µM (mammary) or 0.25–1 µM (gastric). DMSO (0.2%) was used as vehicle control. 48 h after addition of drug, organoids were imaged on the Cytation 5 imaging reader (Biotek): imaging was performed in brightfield and red fluorescence protein (RFP) channels; 4 regions/well, 11 stacks per region. Upon completion of imaging, images were stitched and stacked to produce one merged image per well. Organoid area was calculated in the Cytation software using images from the RFP channel.

### 2.8. Protein Extraction and Western Blotting

Mammary and gastric organoids (WT and *Cdh1*^−/−^) were seeded in 50 µL suspensions of matrigel. One (gastric) or two (mammary) days after seeding, organoids were induced with 0.5 µM endoxifen. On day 5 (gastric) or day 6 (mammary) post seeding, organoids were treated with dasatinib (0.25 µM) or DMSO (0.1%) vehicle for 24 h. On day 6, organoids were harvested using cell recovery solution (Corning) and incubated on ice (with agitation) for 30 min. Cells were then washed with 10 mL of TBS, spun down at 300 rpm (5 min, room temperature) and supernatant removed. The pellets were then frozen at −80 °C overnight. Each pellet was resuspended in 60 µL of lysis buffer (25 mM HEPES, 100 mM NaCl, 1 mM EDTA, 10% (*v*/*v*) glycerol, 1% (*v*/*v*) Triton-X-100) containing Complete Mini Protease Inhibitor tablets (Roche) and incubated on ice for 30 min, vigorously vortexing every 5 min. Tubes were then spun at 13,200 rpm for 10 min at 4 °C and supernatants transferred to fresh tube on ice. Protein was quantified using BCA protein Assay (Thermo Fisher Scientific, Waltham, MA, USA). Protein was diluted with 4× Laemmli buffer and denatured at 95 °C for 5 min. 20 µg of each sample was run on 12% SDS Page gels and transferred onto nitrocellulose membranes. Membranes were blocked for 1 h with 5% BSA-TBST and incubated over night at 4 °C with primary antibodies. Antibody concentrations: pAKT Ser473 (#4060, Cell Signaling, Danvers, MA, USA) at 1:1000, pan AKT at 1:1000 (#4685, Cell Signaling, Danvers, MA, USA), E-cadherin 1:1000 (#AF748, RnD Systems, Minneapolis, MN, USA), P53 1:1500 (#32532, Cell Signaling, Danvers, MA, USA) and B-actin 1:2500 (#A2066, Sigma Aldrich, St. Louis, MO, USA). Fluorescent secondary antibodies anti-rabbit (LI-COR #925-32211), or anti-goat (#925-32214, LI-COR, Lincoln, NE, USA), were both used at 1:10,000. Membranes were imaged on LI-COR Odyssey Imaging System (LI-COR, Lincoln, NE, USA).

## 3. Results

### 3.1. Differential Gene Expression Analysis Results

#### 3.1.1. AKT3 Expression Is Significantly Inversely Correlated with CDH1 Expression in Gastric Tumour Datasets

Previous research in our laboratory demonstrated that *CDH1*-null cells have increased sensitivity to allosteric *AKT* inhibitors (ARQ-092, MK2206) compared to *CDH1*-expressing cells (in isogenic mammary and gastric cancer cell line pairs) [25]. This research also identified a strong negative correlation between *CDH1* and *AKT3* expression in the TCGA STAD dataset (Pearson correlation −0.439, *p*-value < 2.2 × 10^−16^), as well as a weak but non-significant positive correlation between *CDH1* and *AKT1* expression (Pearson correlation 0.081, *p*-value = 0.1). No correlation was found between *AKT2* and *CDH1.* In order to confirm the relationships between *CDH1* and *AKT1*/*AKT3*, we repeated this correlation analysis in a second gastric cancer dataset (GEO accession GSE652254). This analysis confirmed the inverse correlation between *CDH1* and *AKT3* (Pearson correlation −0.429, *p*-value = 6.3 × 10^−15^), and did not find a significant association between *CDH1* and *AKT1* (Pearson correlation 0.07, *p*-value = 0.23) or *AKT2* (Pearson correlation 0.06, *p*-value = 0.32) expression, thus reinforcing the results observed in the TCGA STAD data set.

#### 3.1.2. AKT3-Associated Genes Are Enriched for Extracellular Matrix Pathways

To further understand the activity of *AKT3* in gastric cancer and develop strategies to preferentially target this isoform, we identified *AKT3*-associated genes in the TCGA and GEO datasets. *AKT3*-associated genes were identified by carrying out a differential expression analysis between samples grouped by moderate-to-high (top 66.6%) vs. low (bottom 33.3%) *AKT3* expression. The same process was also carried out to identify *AKT1*-associated genes.

Filtering for significance (FDR adjusted *p*-value < 0.05) produced a list of 7453 AKT1-associated genes, and 6830 AKT3-associated genes. Further filtering for expression fold-change (up- or down-regulation of at least 2-fold) in the AKT3-associated list produced a list of 84 strongly AKT3-associated genes. Genes from the *AKT1*-associated list were then removed from the *AKT3*-associated list to identify *AKT3*-specific associations (Table S2). This resulted in a final list of 51 genes with a significant correlation (all of which were positive) with *AKT3* expression (Table 1 and Table S3). In both the TCGA and GEO data sets, the gene with the strongest statistical association with *AKT3* was discoidin domain receptor tyrosine kinase 2 (*DDR2*), a receptor tyrosine kinase that binds collagen and regulates cell differentiation, migration, proliferation and extracellular matrix remodelling [43]. *DDR2* has also previously been implicated as a driver of peritoneal dissemination and its increased expression is associated with unfavourable clinical characteristics in gastric cancer, such as multiple tumour sites and poor prognosis [44,45]. Other highly correlated genes that have previously been implicated in gastric cancer included *CDH11* [46], *MSRB3* [47] and *FSTL1* [48].

Next, we carried out a Reactome pathway enrichment analysis using the goseq package for R. Of the original 51 genes, 35 were represented in Reactome and were used to identify eight significantly enriched pathways with a Benjamini–Hochberg adjusted *p*-value < 0.05 (Table 2). These pathways included several overlapping ECM-related pathways: non-integrin membrane–ECM interactions, collagen degradation, degradation of the ECM and ECM organisation (Benjamini–Hochberg adjusted *p*-values of 0.045, 0.025, 0.0045, and 1.4 × 10^−6^, respectively). Together, the identification of these ECM pathways and the co-expression of *AKT3* and *DDR2* in E-cadherin-low gastric tumours suggest an active link between ECM remodelling and AKT3 signalling in DGC.

### 3.2. Breast Cells Lacking CDH1 Are More Sensitive to the Kinase Inhibitor Dasatinib Than Wild-Type Cells

The identification of DDR2 as associated with AKT3 suggested that inhibition of this collagen-activated kinase may provide a means to more specifically target the AKT3 isoform than is possible with pan AKT inhibitors. To assess kinase inhibitors with activity against DDR2, we first utilised a previously characterised pair of isogenic cell lines: MCF10A-WT (containing wild type *CDH1*) and MCF10A-*CDH1*^−/−^ (containing a homozygous deletion in the *CDH1* locus) [49]. We initially tested four small molecule inhibitors: imatinib, ponatinib, nilotinib and dasatinib. These four compounds were developed as inhibitors to BCR-ABL for the treatment of chronic myeloid leukaemia, but have since been found to have a number of novel kinase and non-kinase targets [50], including SRC and DDR2 [51,52].

**Figure 1:**
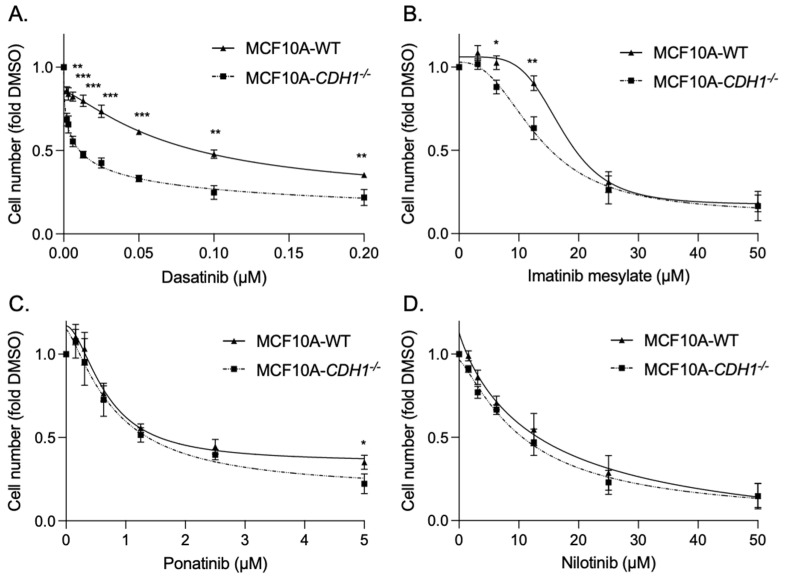
Mammary epithelial cells lacking *CDH1* expression are preferentially sensitive to the cytotoxic effects of dasatinib. (**A**–**D**) Normalised MCF10a-WT and *CDH1*^−/−^ cell counts 48 h after treatment with serial dilutions of dasatinib (**A**), imatinib mesylate (**B**), ponatinib (**C**) and nilotinib (**D**). Wild-type, black bars, *CDH1*^−/−^ grey bars. Six fields per well at 4× magnification were captured using the Cytation 5 imager (Biotek). Nuclei were counted using Gen5 (Biotek) and normalised to the vehicle control for each cell line. (For all graphs, error bars = SEM; * *p* < 0.05, ** *p* < 0.01 and *** *p* <0.001; *n* ≥ 3 independent biological replicates; unpaired two-sided *t*-test).

After 48 h of treatment, MCF10A-*CDH1*^−/−^ cells exhibited a greater to sensitivity to two of the inhibitors tested: dasatinib, at all concentrations tested (WT EC50 0.1 µM ± 0.0004 vs. *CDH1*^−/−^ EC50 0.01 µM ± 0.0004, *p* = 0.00005), and to a lesser extent, imatinib (WT EC50 10.6 µM ± 1.0 vs. *CDH1*^−/−^ EC50 15.8 µM ± 1.2, *p* = 0.04) identifying a vulnerability in *CDH1*-null cells to drugs which inhibit kinases including DDR2, SRC and BCR-ABL (Figure 1A,B). No significant difference in growth was seen after treatment with nilotinib and ponatinib (Figure 1C,D). MCF10A- *CDH1*^−/−^ cells also exhibited a greater sensitivity to a commercially available selective DDR2 allosteric inhibitor—WRG-028 (WT EC50 13.7 µM ± 0.2 vs. *CDH1*^−/−^ EC50 6.8 µM ± 0.1, *p* = 0.0009), indicating that the SL effect seen in *CDH1*^−/−^ cells is at least in part due to the inhibition of DDR2 activity [53] (Figure S1). Interestingly, dasatinib exhibited an opposite effect in NCI-N87 gastric cancer cells deficient in E-cadherin (N87-*CDH1*^−/−^), with these cells having superior survival after treatment compared to WT cells (N87-WT) (Figure S2). This is presumably explained by genes that are upregulated in NCI-N87 cells following E-cadherin loss which can compensate for the vulnerability observed in the non-malignant MCF10A E-cadherin-deficient cells [54]. Ultimately the identification of these genes may inform the identification of dasatinib resistance pathways and elucidate the identification of robust drug combinations for the treatment of advanced DGC and LBC.

**Figure 2:**
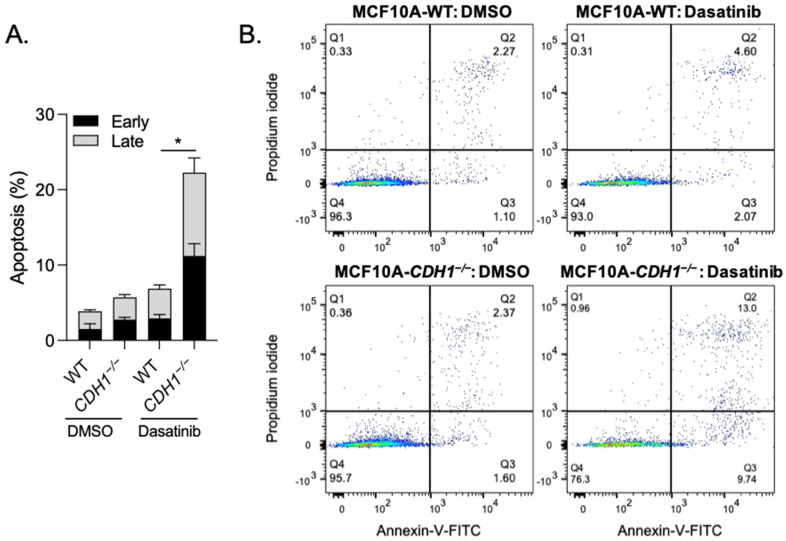
Dasatinib induces apoptosis in mammary cells lacking E-cadherin. (**A**) Total apoptosis (Annexin-V-FITC and propidium iodide positive cells) (early + late apoptosis) detected by flow cytometry after 72 h drug treatment. (**B**) Representative histograms of MCF10A-WT and *CDH1*^−/−^ cells stained with Annexin-V-FITC and propidium iodide and analysed on BD Fortessa flow cytometer. Q4; live cells, Q3; early apoptotic cells and Q2; late apoptotic cells. (For bar graph, error bars = SEM; * *p* < 0.05; *n* ≥ 3 independent biological replicates; unpaired two-sided *t*-test).

We next examined whether induction of apoptotic cell death could account for the significantly reduced MCF10A-*CDH1*^−/−^ cell number after treatment with dasatinib. We measured apoptosis by flow cytometry analysis of Annexin-V-FITC/propidium iodide-stained cells after 48 h treatment with 0.2 µM dasatinib (or DMSO controls). Importantly, basal apoptosis levels between MCF10A-WT and *CDH1*^−/−^ cells were not significantly different (Figure 2A). After dasatinib treatment, total apoptosis levels in MCF10A-WT cells was increased by 2.4-fold compared to DMSO controls (DMSO 2.8% vs. dasatinib 6.8%) (Figure 2A,B). However, in MCF10A-*CDH1*^−/−^ cells, treatment with dasatinib significantly increased total apoptosis from 4.5% (in DMSO controls) to 20.0%, a fold change of 4.4. Increases in both early (Annexin-V-FITC single staining) and late apoptosis (double staining with Annexin-V-FITC/propidium iodide) equally accounted for the difference in total apoptosis between the two cell lines (Figure 2A). These results led us to conclude that dasatinib preferentially induces apoptosis in MCF10A-*CDH1*^−/−^ cells and that the synthetic lethal effect observed is at least in part due to preferential cytotoxicity in cells lacking *CDH1*.

### 3.3. Conditional Knock-Out of Cdh1 Sensitises Gastric and Mammary Organoids to Dasatinib

**Figure 3:**
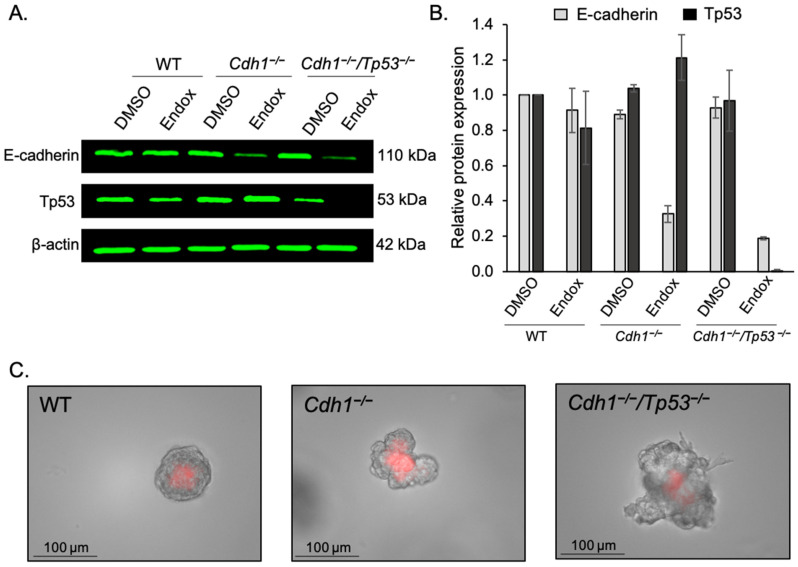
Characterisation of mammary organoids. (**A**) Endoxifen (endox) mediated knockout of E-cadherin and/or Tp53 in mammary organoids was detected utilizing western blotting. (**B**) Relative expression of E-cadherin and Tp53 protein in WT, *Cdh1*^−/−^ and *Cdh1*^−/−^*Tp53*^−/−^ mammary organoids. (**C**) 20× Brightfield and RFP channel images of mammary organoids induced with endoxifen.

To address the limitations of using a 2D model system for drug testing, especially considering DDR2 is activated by the extracellular ligand collagen, we tested dasatinib in mouse-derived mammary and gastric organoids. Organoids are 3D cellular cultures of stem cell origin, typically obtained through mechanical and enzymatic disruption of tissues and grown in gels made of complex protein mixtures (including ECM proteins such as collagen and laminin). In our organoid models, mammary and stomach glands were derived from transgenic mice containing a construct of CD44-Cre/*Cdh1*^(^^fl/fl)^/tdTomato ^(fl/fl)^. Upon treatment with endoxifen, Cre recombinase activity is induced, resulting in excision of exons 6 to 10 of the *Cdh1* gene, abrogating E-cadherin activity. Simultaneously, a premature stop codon is removed from the tdTomato construct, leading to expression of the red fluorescent protein tdTomato. CD44-Cre/*Cdh1*^(Wt/Wt)^/tdTomato^(fl/fl)^ mice were used as controls (referred to as WT), where induction with endoxifen results in tdTomato expression but does not affect Cdh1. An additional mouse strain containing a construct of CD44/Cre/ *Cdh1*^(^^fl/fl^^)^/*Tp53*^(^^fl/fl^^)^/tdTomato ^(^^fl/fl^^)^, which upon induction leads to the simultaneous knockout of E-cadherin and Tp53 expression in CD44-expressing cells was used to generate organoids that modelled more advanced DGC. Depletion of Tp53 and/or E-cadherin protein in our organoid models was confirmed by western blot for the mammary organoids (Figure 3A,B and Figure S5) and western blot/confocal microscopy for the gastric organoids [24]. Brightfield imaging clearly shows the distinct morphological changes induced by knockout of *Cdh1* and *Tp53* in mammary organoids (Figure 3C). WT mammary organoids exhibit a smooth rounded morphology, however, upon depletion of E-cadherin, these organoids tended to grow larger and display a more amorphous and irregular morphology. Following knockout of both *Cdh1* and *Tp53*, the growth of mammary organoids accelerated markedly, and morphology became significantly more irregular, with the appearance of cellular projections from the main body of the organoids, suggestive of a more invasive phenotype. This is consistent with the effect of *Tp53* knockout seen in other organoid models, where loss of *Tp53* alone is sufficient to confer metastatic ability to mouse intestinal organoids in a spleen transplantation model [55].

**Figure 4:**
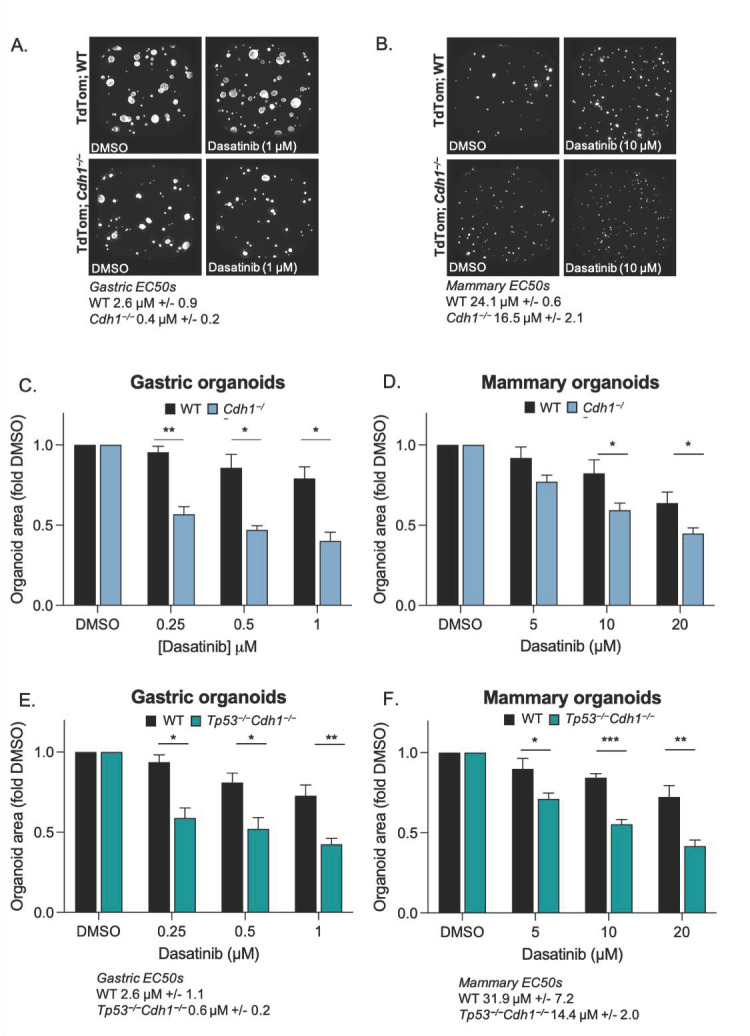
Mouse-derived organoids containing *Cdh1*^−/−^ cells are more sensitive to the growth inhibiting effects of dasatinib. Representative photos of WT and *Cdh1*^−/−^ gastric (**A**) and mammary (**B**) organoids after 48 h treatment with DMSO or dasatinib. Bar graphs showing relative area of DMSO or dasatinib treated gastric (**C**) or mammary (**D**) organoids. Bar graphs showing relative area of DMSO or dasatinib treated WT or *Tp53*^−/−^*Cdh1*^−/−^ gastric (**E**) and mammary (**F**) organoids. (For all graphs, error bars = SEM; * *p* < 0.05, ** *p* < 0.01 and *** *p* <0.001; *n* ≥ 3 independent biological replicates; unpaired two-sided *t*-test).

The main measure of dasatinib activity in these organoid models was average area calculated 48 h after drugging. DMSO (vehicle) treated gastric and mammary WT and *Cdh1*^−/−^ organoids displayed normal growth and no morphological evidence of cell death (Figure 4A,B). However, after 48 h treatment with 1 µM dasatinib, *Cdh1*^−/−^ gastric organoids were 0.4-fold the size of DMSO controls, whereas the WT organoids were 0.8-fold smaller, a difference of 39% between WT and *Cdh1*^−/−^ organoids at this concentration of drug (Figure 4C,D).

Interestingly, mammary organoids (WT and *Cdh1*^−/−^) were less sensitive than gastric organoids to dasatinib (i.e., mammary WT EC50 = 24.1 µM vs. gastric WT EC50 = 2.6 µM) (Figure 4B,D). Similar to gastric organoids, *Cdh1*^−/−^ mammary organoids were more sensitive than WT to dasatinib, and after 48 h treatment with 10 µM dasatinib, the average total area of *Cdh1*^−/−^ mammary organoids was 0.6-fold of DMSO controls vs. WT 0.8-fold of controls, a difference of 23% between WT and *Cdh1*^−/−^ organoids at this concentration of drug (Figure 4B,D).

By way of comparison, we also tested the pan-AKT allosteric inhibitors MK2206 and ARQ-092 on these organoid models. After treatment with 3.1 µM MK2206, the average total area of *Cdh1*^−/−^ gastric organoids was 0.5-fold of DMSO controls vs. WT 0.8-fold of controls, a difference of 27% between WT and *Cdh1*^−/−^ organoids at this concentration of drug (Figure S3A). After treatment with 12.5 µM MK2206, the average total area of *Cdh1*^−/−^ mammary organoids was 0.4-fold of DMSO controls vs. WT 0.8-fold of controls, a difference of 41% between WT and *Cdh1*^−/−^ organoids (Figure S3B). The average total area of *Cdh1*^−/−^ gastric organoids after treatment with 0.25 µM ARQ-092 was 0.6-fold of DMSO controls vs. WT 0.9-fold of controls, a difference of 34% between WT and *Cdh1*^−/−^ organoids (Figure S3C). Similarly, after treatment with 2.5 µM ARQ-092, the average total area of *Cdh1*^−/−^ mammary organoids was 0.4-fold of DMSO controls vs. WT 0.9-fold of controls, a difference of 44% between WT and *Cdh1*^−/−^ organoids (Figure S3D). Similar to dasatinib treatment, gastric organoids exhibited a greater sensitivity to the AKT inhibitors than mammary organoids.

Next, we tested the effect of dasatinib on mammary and gastric organoids lacking both *Cdh1* and *Tp53* expression. These organoids were generated in order to more closely reflect the genetic complexity of advanced DGC and LBC which have *Tp53* mutation frequencies of ~33% and ~7–18%, respectively [56,57,58]. The difference in the response of *Tp53*^−/−^*Cdh1*^−/−^ gastric and mammary organoids to dasatinib was similar to that of *Cdh1*^−/−^ organoids (Figure 4E,F). After treatment with 1 µM dasatinib, *Tp53*^−/−^*Cdh1*^−/−^ gastric organoids were 0.4-fold the size of DMSO controls, whereas the WT organoids were 0.7-fold (Figure 4E). After treatment with 10 µM dasatinib, *Tp53*^−/−^*Cdh1*^−/−^ mammary organoids were 0.55-fold the size of DMSO controls, whereas the WT organoids were 0.8-fold (Figure 4F). This indicates that the addition of the loss of *Tp53* does not interfere with the observed synthetic lethal effect and that dasatinib may be an option not only for the chemoprevention of DGC and LBC in germline *CDH1* mutation carriers, but also the treatment of a subset of advanced tumours harbouring both *CDH1* and *TP53* mutations.

### 3.4. Dasatinib Inhibits AKT Phosphorylation in Cdh1-Deficient Organoids

**Figure 5:**
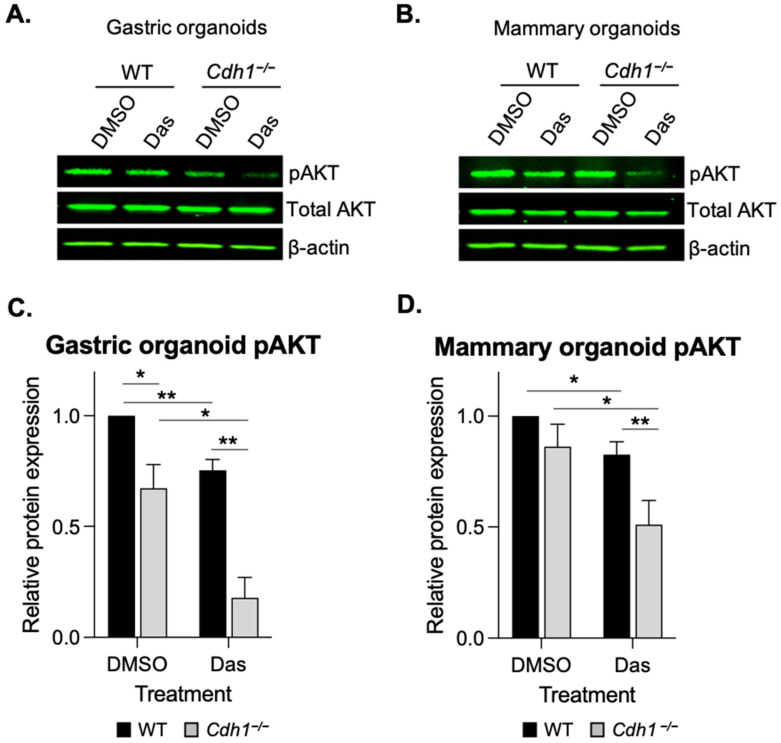
Dasatinib preferentially inhibits pAKT in mouse derived organoids lacking *Cdh1*^−/−^. (**A**,**B**) Western blots of pAKT-Ser473 and total AKT levels in gastric (**A**) and mammary (**B**) organoids treated with DMSO or dasatinib (0.5 µM) for 24 h. (**C**,**D**) Relative expression of pAKT gastric (**C**) and mammary (**D**) organoids. (For all graphs, error bars = SEM; * *p* < 0.05, ** *p* < 0.01; *n* ≥ 3 independent biological replicates; unpaired two-sided *t*-test).

**Figure 6:**
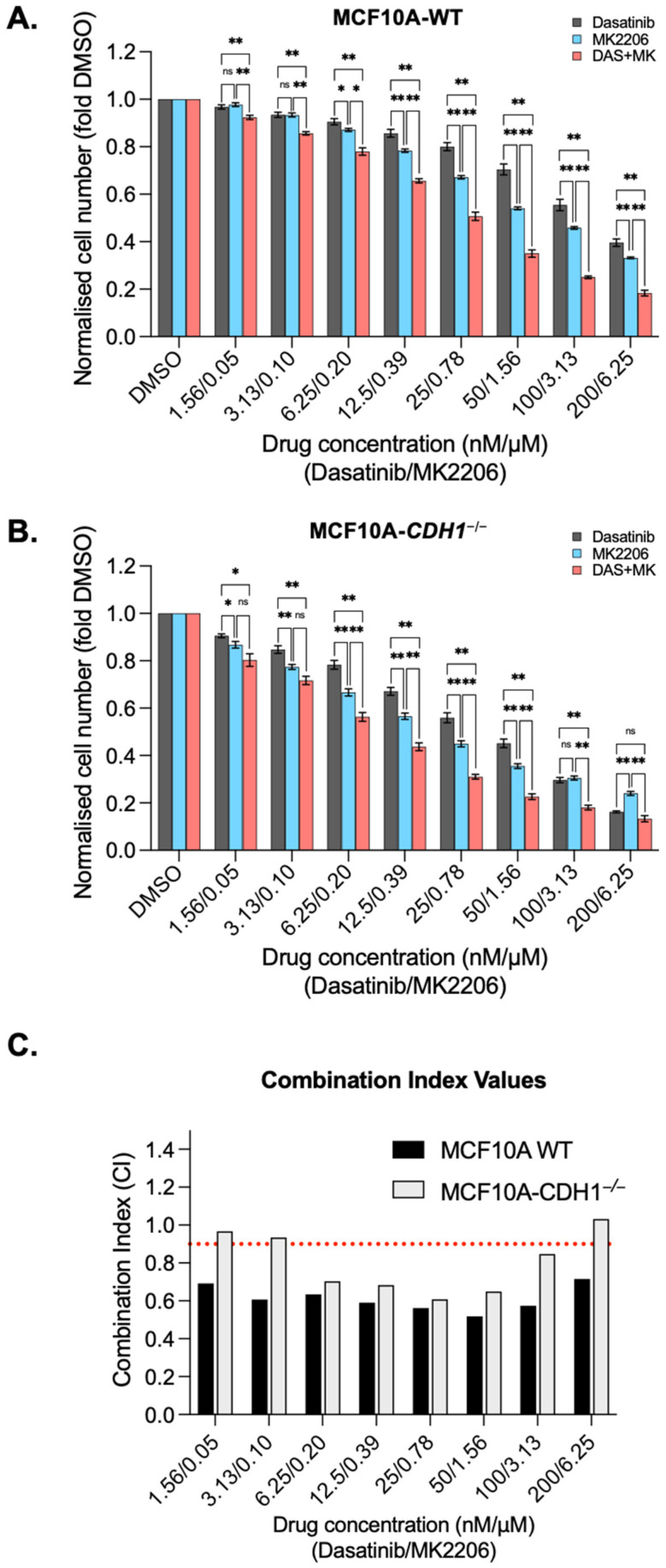
Combining dasatinib with the allosteric AKT inhibitor MK2206 is synergistic in MCF10A cells. Normalised MCF10A-WT (**A**) and *CDH1*^−/−^ (**B**) cell counts 48 h after treatment with serial dilutions of dasatinib, MK2206 or a combination of dasatinib and MK2206. (**C**) Combination index (CI) values for MCF10A-WT and *CDH1*^−/−^ cells treated with combination of dasatinib and MK2206. Values below 0.9 indicate the drug combination is synergistic at that concentration. For all graphs, error bars = SEM; ns: *p* > 0.05, * *p* < 0.05, ** *p* < 0.01; *n* ≥ 3 independent biological replicates; unpaired two-sided *t*-test).

To determine if the synthetic lethal relationship between E-cadherin and dasatinib treatment in organoids was at least in part due to drug-induced alterations in the pro-survival AKT cellular signalling pathway, we measured the levels of phosphorylated AKT (pAKT-Ser473) after dasatinib treatment in the WT and *Cdh1*^−/−^ gastric and mammary organoids. Interestingly, levels of pAKT were significantly lower (19%) in DMSO treated *Cdh1*^−/−^ gastric organoids compared with WT (Figure 5A,C and Figure S6). pAKT levels were reduced in both WT and *Cdh1*^−/−^ gastric organoids after 24 h of dasatinib treatment, by 25% in WT and 63% in *Cdh1*^−/−^ (Figure 6A,C). There was no significant difference in the levels of pAKT and total AKT in DMSO treated WT and *Cdh1*^−/−^ mammary organoids (Figure 5B,D), however, after treatment with 0.25 µM dasatinib for 24 h, pAKT was preferentially reduced in *Cdh1*^−/−^ mammary organoids (52% reduction vs. 17% reduction in WT organoids) (Figure 5B,D and Figure S7). Taken together, these results indicate that dasatinib inhibits AKT signalling within both WT/*Cdh1*^−/−^ mammary and gastric organoids, however the effect is significantly more pronounced in *Cdh1*^−/−^ organoids.

### 3.5. Dasatinib Is Synergistic with the Allosteric AKT Inhibitor MK2206 in MCF10A Cells

After establishing the synthetic lethal effects of dasatinib, it was next assessed in combination treatment with both the pan-AKT inhibitor MK2206 and the SRC family kinase inhibitor PP1, with the aim of evaluating these combinations for synergistic effects. Synergy could provide an opportunity to reduce doses in future clinical applications, potentially reducing the risk of dose limiting toxicities and improving longer term tolerability. Both MCF10A-WT and MCF10A-*CDH1*^−/−^ cells exhibited greater sensitivity to combinations of dasatinib and MK2206 than the single agents (Figure 6A,B) at most concentrations tested. A combination index (CI) value was calculated at each treatment concentration for the combinations, with values less than 0.9 considered synergistic, and those above 1.1 antagonistic [59]. The CI values demonstrate that combination treatment of dasatinib/MK2206 is synergistic in both WT and *CDH1*^−/−^ cells from 6.3 nM /0.2 µM (Dasatinib/MK2206) to 100 nM/3.1 µM treatments (Figure 6C).

Combination treatment with dasatinib and PP1 produced a significant inhibition of cell growth compared to the inhibition seen in either single agent in the highest four concentrations in WT cells and at the three highest concentrations in *CDH1*^−/−^ cells (Figure S4A,B). However, the reduction in cell number seen with combination treatment was only synergistic in WT cells, with highly synergistic CI at the two highest combination concentrations tested (0.5 and 0.4, respectively). (Figure S4C). Interestingly, combination treatment with dasatinib/PP1 appeared to be markedly antagonistic in WT cells at mid-ranges, with CI values of 2.9 (at 6.25 nM /1.25 µM dasatinib/PP1) and 2.1 (at 12.5 nM/2.5 µM dasatinib/PP1) (Figure S4C). While significant reductions in *CDH1*^−/−^ cell number were achieved at selected concentrations using this combination, due to the effect of the individual drugs in this cell line, this combination demonstrated an additive (CI 0.9–1.1) interaction at most concentrations tested. (Figure S4C). The difference in synergy between the two drug combinations tested may reflect the extent of overlap between the respective targets of each drug combination, with the synthetic lethal targets of dasatinib/PP1 likely to be more overlapped than dasatinib/MK2206. While there is potential for dasatinib-AKT inhibitor combinations to be useful clinically, the similar level of synergy in both WT and *CDH1*^−/−^ cells suggests that this combination may not increase the available therapeutic index between normal cells and early *CDH1*-null cancers.

## 4. Discussion

We have previously shown that *AKT3*, but not *AKT1* or *AKT2*, is upregulated in gastric cancers with low *CDH1* expression and have identified an E-cadherin-AKT synthetic lethal relationship [25]. Since isoform-specific AKT inhibitors are not yet available, we have aimed to identify non-direct routes to specifically target AKT3 signalling in E-cadherin-deficient cells. Through differential expression and Reactome pathway analysis, we identified ECM remodelling pathways as potential upstream regulators of *AKT3*. The most strongly associated gene was the tyrosine kinase receptor *DDR2*, leading us to test the nanomolar range inhibitor dasatinib in our E-cadherin-null models [51].

In normal cells, DDR2 is a receptor for collagen (preferentially fibrillar collagens and the non-fibrillar collagens II and X [43,60]) and is involved in the transduction of cues from the binding of ECM collagens in order to activate signalling networks that support normal cellular function [43]. Functionally, DDR2 activity and downstream signalling has been associated with cell proliferation, survival, adhesion, spreading and differentiation [60]. Activated DDR2 has been shown to signal through a number of pathways including ERK, MAPK, PI3K and possibly FAK [61,62] Induction of EMT in a variety of model systems has been demonstrated to correlate with a switch from the expression of the more epithelial DDR1 to the more mesenchymal DDR2 [60,63]. In a human mammary epithelial cell line, the expression of EMT inducers, such as Snail, Slug and TGF-ß induced an “EMT core signature” which included an upregulation of DDR2 expression concomitant to a down regulation of DDR1 [64]. Of particular interest, DDR2 has been demonstrated to stabilise Snail1 protein in breast cells and a significant association Snail1, lack of E-cadherin and presence of DDR2 expression was observed in samples of human breast cancer [65]. Snail is a key regulator of EMT, and its expression represses E-cadherin [66]. Deregulation of DDR2 expression and/or signalling has been associated with a wide variety of cancers including lung cancer, breast cancer, lymphoma and leukaemia [60]. In addition, DDR2 has previously been implicated as a driver of gastric cancer dissemination [44]. As a plasma membrane bound receptor, DDR2 presents as an attractive therapeutic target for a variety of cancers, inflammatory conditions, as well as brain and renal disease [67].

As dasatinib is not exclusively an inhibitor of DDR2 (also exhibiting a well characterised inhibition of BCR-ABL and SRC) it is possible that a combination of dasatinib targets is contributing to the synthetic lethal effect seen in our E-cadherin-deficient models. Inhibition of SRC family kinases is of particular interest as we have previously seen a synthetic lethal effect with the SRC family inhibitors saracatinib, PP1 and PP2 in MCF10A cells lacking *CDH1* [23,68]. SRC and DDR2 are intimately linked, with SRC associating with, and phosphorylating, DDR2 on tyrosine residues (in an activation loop) after collagen binding. This leads to DDR2 activity and additional association with the adaptor molecule Shc [69,70]. Shc proteins typically exert their effects via activation of MAPK and PI3K/AKT signalling pathways [71]. SRC can also directly interact with AKT and phosphorylate tyrosine residues [72] leading to optimal activation of AKT in vitro and in vivo [73]. Interestingly, dual inhibition of SRC and DDR2 with a novel small molecule drug has been shown to lead to superior growth inhibition (compared with inhibition of DDR2 alone) in lung cells harbouring DDR2 mutations [74].

In this study, treatment with dasatinib led to a reduction in phosphorylated AKT in both WT and *Cdh1*^−/−^ organoids (mammary and gastric), but the inhibitory effect was significantly more pronounced in E-cadherin-deficient organoids. This indicates that inhibition of AKT signalling contributes to the synthetic lethal effect of dasatinib in our E-cadherin-deficient models, although other downstream pathways that are regulated by the SRC family, including FAK and MAPK signalling, are also likely to be involved.

When combined with dasatinib in MCF10A-WT and *CDH1*^−/−^ cells, MK2206 exhibited synergy, indicating that the simultaneous targeting of SRC, DDR2 and AKT may be effective in specifically targeting E-cadherin-deficient cancer cells. However, because this synergy was also seen in WT cells, this particular drug combination may not increase the therapeutic window. To achieve clinical utility for HDGC chemoprevention, a combination which is more synergistic in *CDH1*-null cells compared to WT across all practical concentrations is likely to be necessary. We also tested dasatinib in combination with PP1, a reversible, ATP-competitive and selective inhibitor of the SRC family of protein tyrosine kinases. This combination was neither significantly synergistic nor antagonistic in *CDH1*^−/−^ cells, suggesting that this drug combination will not be useful for targeting E-cadherin-deficient cancers. Since both dasatinib and PP1 inhibit SRC, this may indicate that future selection of combinations could benefit from combining drugs which target distinct vulnerabilities in *CDH1*^−/−^ cells.

Dasatinib is currently approved for the treatment of imatinib-resistant or intolerant chronic myeloid leukaemia and Philadelphia-positive acute lymphoblastic leukaemia [75]. However, there are a number of clinical trials underway to evaluate it in solid tumours (clinicaltrials.gov accessed on 20 January 2022). Dasatinib causes several adverse events, such as anaemia, thrombocytopenia, pleural effusion/fluid retention, GI disorders, skin rashes, fatigue and diarrhoea [75]. Although these side effects are manageable, we anticipate that significantly less toxicity will be a requirement for chemoprevention strategies for E-cadherin-null cancers. This may be achieved simply through lower dose (enabled by the low clinical urgency that exists in a chemoprevention setting) or synergistic combinations.

Alternative strategies for drug delivery systems will also contribute to lessening treatment toxicity. In the context of LBC chemoprevention, transdermal delivery of treatments may be an effective approach. The breast is a promising tissue to benefit from transdermal therapies due to its embryonic origin as a skin appendage, well developed internal lymphatic and venous circulation to aid in drug circulation within the breast and the presence of fat layers to aid in localised drug accumulation [76,77,78]. To date, transdermal delivery of chemopreventative drugs (such as the tamoxifen metabolite 4-hydroxytamoxifen and chemotherapeutic agents (including dasatinib and docetaxel) have been extensively explored in murine models with promising results [79,80]. Additionally, human clinical studies have confirmed this mode of drug delivery leads to a sharp restriction in plasma levels of the drugs, which would most likely reduce systemic side effects, as well as having drug distribution within the breast equivalent to oral dosing [81,82]. One of the challenges with local transdermal delivery of drugs is the properties of the drugs themselves, with some drugs not readily permeating the skin. This may be overcome using different gel formulations, the binding of drugs to more soluble factors, or incorporation of the drugs into micellar nanostructures [79,80,83]. Comparable delivery systems that deliver and retain drugs in close proximity to the gastric mucosa could also be used to minimise systemic toxicity during chemoprevention. For example, release of drug only in the acidic environment of the stomach, combined with a gastro-retentive formulation to mitigate the effect of gastric emptying, would lead to increased drug delivery at the target site and reduce off-target effects during repeated drug administration.

## 5. Conclusions

We have identified that breast cells and mouse-derived organoids (mammary and gastric) that are deficient in E-cadherin have increased sensitivity to the multi-kinase inhibitor dasatinib. This effect is likely to be at least partly attributable to dasatinib’s inhibition of DDR2 and SRC, and indirect effects on AKT3 signalling. The identification of the synthetic lethal relationship between E-cadherin and dasatinib indicates that inhibition of the DDR2-SRC-AKT3 axis may be a novel strategy for the treatment of hereditary and sporadic cancers with mutations in the *CDH1* gene.

## Figures and Tables

**Table 1:** Top 20 *AKT3*-associated genes after significance and fold-change filtering (FDR adjusted *p*-value < 0.05 and at least 2-fold up- or down-regulation) in the TCGA and GEO datasets. Expression fold change (FC) and FDR-adjusted *p*-value are shown per gene, for each data set. Genes are sorted based on the average rank of the adjusted *p*-value across the two data sets. The full list of 51 *AKT3*-specific genes is provided in Table S3.

Gene	TCGA FC	GEO FC	TCGA Adj *p*-Value	GEO Adj *p*-Value
*DDR2*	5.13	2.04	6.84 × 10^−51^	2.89 × 10^−35^
*CDH11*	3.14	2.33	1.99 × 10^−43^	1.26 × 10^−31^
*AOC3*	4.65	2.45	2.91 × 10^−43^	1.57 × 10^−30^
*MSRB3*	4.12	2.06	9.74 × 10^−43^	6.82 × 10^−30^
*FSTL1*	2.61	2.07	9.96 × 10^−43^	4.76 × 10^−29^
*DPYSL3*	4.28	2.07	4.95 × 10^−42^	1.46 × 10^−28^
*COL8A1*	5.33	2.02	8.41 × 10^−42^	9.60 × 10^−28^
*TIMP3*	3.16	2.16	9.55 × 10^−40^	1.96 × 10^−27^
*CCDC80*	5.29	2.28	9.89 × 10^−39^	5.48 × 10^−27^
*LTBP1*	3.00	2.10	1.54 × 10^−38^	4.74 × 10^−26^
*PDLIM3*	4.40	2.50	2.10 × 10^−38^	1.85 × 10^−25^
*C1S*	2.87	2.37	3.61 × 10^−36^	2.66 × 10^−25^
*GREM1*	6.27	2.35	9.83 × 10^−36^	3.64 × 10^−25^
*GLT8D2*	2.78	2.18	2.28 × 10^−35^	1.88 × 10^−24^
*GAS1*	5.25	3.11	2.37 × 10^−35^	2.77 × 10^−24^
*TNS1*	3.67	2.35	5.32 × 10^−35^	5.75 × 10^−24^
*KCNMA1*	7.31	3.77	3.20 × 10^−34^	1.16 × 10^−23^
*MYLK*	4.59	2.61	3.98 × 10^−34^	1.31 × 10^−23^
*C1R*	2.76	2.14	6.15 × 10^−34^	2.33 × 10^−23^
*ADAMTS1*	2.75	2.08	1.82 × 10^−33^	3.75 × 10^−23^

**Table 2:** Reactome pathways significantly enriched (Benjamini–Hochberg-adjusted *p*-value < 0.05) within a list of 35 *AKT3*-associated genes. Shown from left to right are Reactome pathway name, Benjamini–Hochberg-adjusted *p*-value, symbols for genes differentially expressed in the dataset by pathway, the number of genes differentially expressed in the dataset by pathway and the total number of genes within the Reactome database for each pathway.

Reactome Pathway	Adj *p*-Value	Differentially Expressed Genes in Pathway	No. of Differentially Expressed Genes in Pathway	Total No. of Genes in Pathway
Extracellular matrix organization	1.40 × 10^−6^	*COL6A3*, *COL8A1*, *COL15A1*, *FBLN1*, *FN1*, *TNC*, *LTBP1*, *MMP2*, *DDR2*, *ASPN*, *SDC2*, *ADAMTS1*	12	289
Regulation of IGF transport and uptake by IGFBPs	1.00 × 10^−4^	*FSTL1*, *FN1*, *TNC*, *IGFBP5*, *LTBP1*, *MMP2*, *SDC2*	7	120
Smooth Muscle Contraction	1.00 × 10^−4^	*MYH11*, *MYLK*, *ACTA2*, *ACTG2*, *CALD1*	5	38
Post-translational protein phosphorylation	0.00058	*FSTL1*, *FN1*, *TNC*, *IGFBP5*, *LTBP1*, *SDC2*	6	103
Muscle contraction	0.0022	*MYH11*, *MYLK*, *PLN*, *ACTA2*, *ACTG2*, *VIM*, *CALD1*	7	194
Degradation of the extracellular matrix	0.0045	*COL6A3*, *COL8A1*, *COL15A1*, *FN1*, *MMP2*, *ADAMTS1*	6	133
Collagen degradation	0.025	*COL6A3*, *COL8A1*, *COL15A1*, *MMP2*	4	59
Non-integrin membrane-ECM interactions	0.045	*FN1*, *TNC*, *DDR2*, *SDC2*	4	59

## Data Availability

The data presented in this study are available on request from the corresponding author.

## Supplementary Materials

The following are available online at https://www.mdpi.com/article/10.3390/cancers14071609/s1, Table S1: Organoid media components, Table S2: Genes that were associated with both *AKT1* and *AKT3*, Table S3: The 51 significantly *AKT3* associated genes with a fold change in expression >2 between high and low *AKT3* expressing samples in both the GEO and TCGA datasets. Figure S1. Treatment with the DDR2 inhibitor WRG-028 is synthetic lethal in E-cadherin deficient mammary epithelial cells. Figure S2. N87 gastric cells lacking *CDH1* are less sensitive to dasatinib than WT cells. Figure S3. Mouse derived organoids containing *Cdh1−/−* cells are more sensitive to the growth inhibiting effects of allosteric AKT inhibitors. Figure S4. Combining dasatinib with the SRC inhibitor PP1 is synergistic in MCF10A cells at higher concentrations, Figure S5. Uncropped western blots: mammary organoid induction test, Figure S6. Uncropped western blots: gastric organoids +/− dasatinib, Figure S7. Uncropped western blots: mammary organoids +/− dasatinib.
