# Supplementary material for: E-Cadherin-Deficient Cells Are Sensitive to the Multikinase Inhibitor Dasatinib
Source: Cancers (Basel). 2022 Mar 22;14(7):1609. doi: 10.3390/cancers14071609 (PMC8996982; doi:10.3390/cancers14071609)
Supplement: Supplementary file 1 [file cancers-14-01609-s001.zip › cancers-1599681-supplementary.pdf]

Supplementary Materials

E-Cadherin-Deficient Cells Are Sensitive to the Multikinase Inhibitor Dasatinib

Nicola Bougen-Zhukov, Lyvianne Decourtye-Espiard, Wilson Mitchell, Kieran Redpath, Jacqui Perkinson, Tanis Godwin, Michael A. Black and Parry Guilford

Centre for Translational Cancer Research (Te Aho Matatū), Cancer Genetics Laboratory, Department of Biochemistry, University of Otago, Dunedin 9016, New Zealand; nicola.bougen-zhukov@otago.ac.nz (N.B.-Z.); lyvianne.decourtye@otago.ac.nz (L.D.-E.); mitwi509@student.otago.ac.nz (W.M.); redki406@student.otago.ac.nz (K.R.); jacquiperkinson96@gmail.com (J.P.); tanis.godwin@otago.ac.nz (T.G.); mik.black@otago.ac.nz (M.A.B.)
* Correspondence: parry.guilford@otago.ac.nz; Tel.: +64-3-479-7673

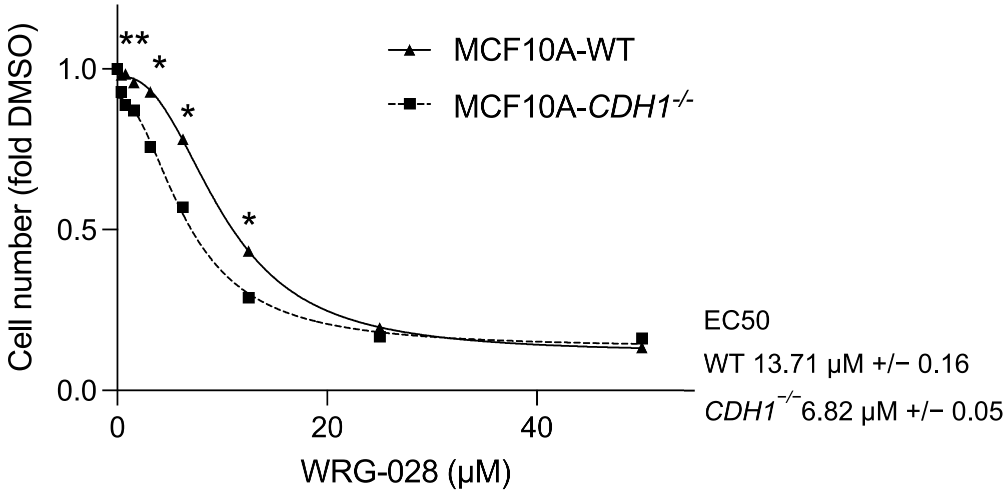

Figure S1. Treatment with the DDR2 inhibitor WRG-028 is synthetic lethal in E-cadherin deficient mammary epithelial cells. (For all graphs, error bars = SEM; * $p < 0.05$, ** $p < 0.01$; $n \geq 3$ independent biological replicates; unpaired two-sided *t*-test.).

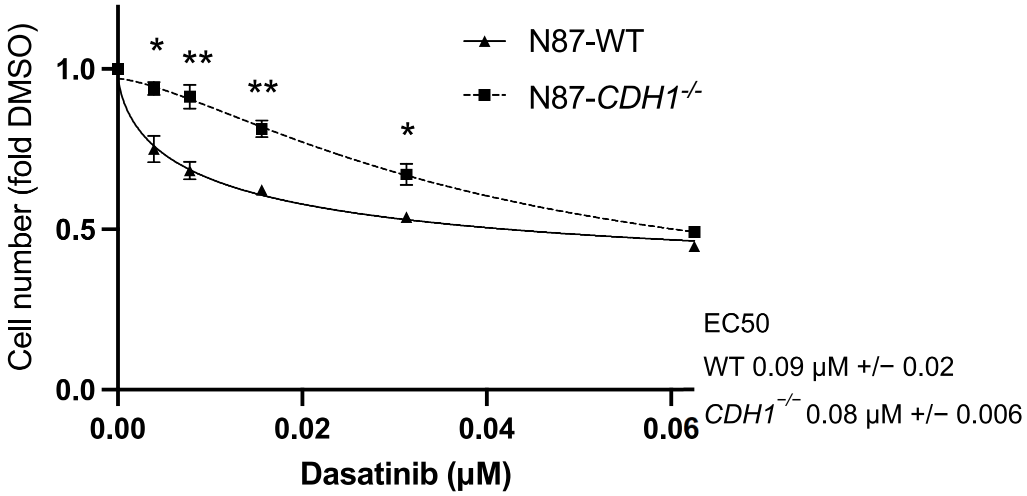

Figure S2. N87 gastric cells lacking *CDH1* are less sensitive to dasatinib than WT cells. (For all graphs, error bars = SEM; * $p < 0.05$, ** $p < 0.01$; $n \geq 3$ independent biological replicates; unpaired two-sided *t*-test.).

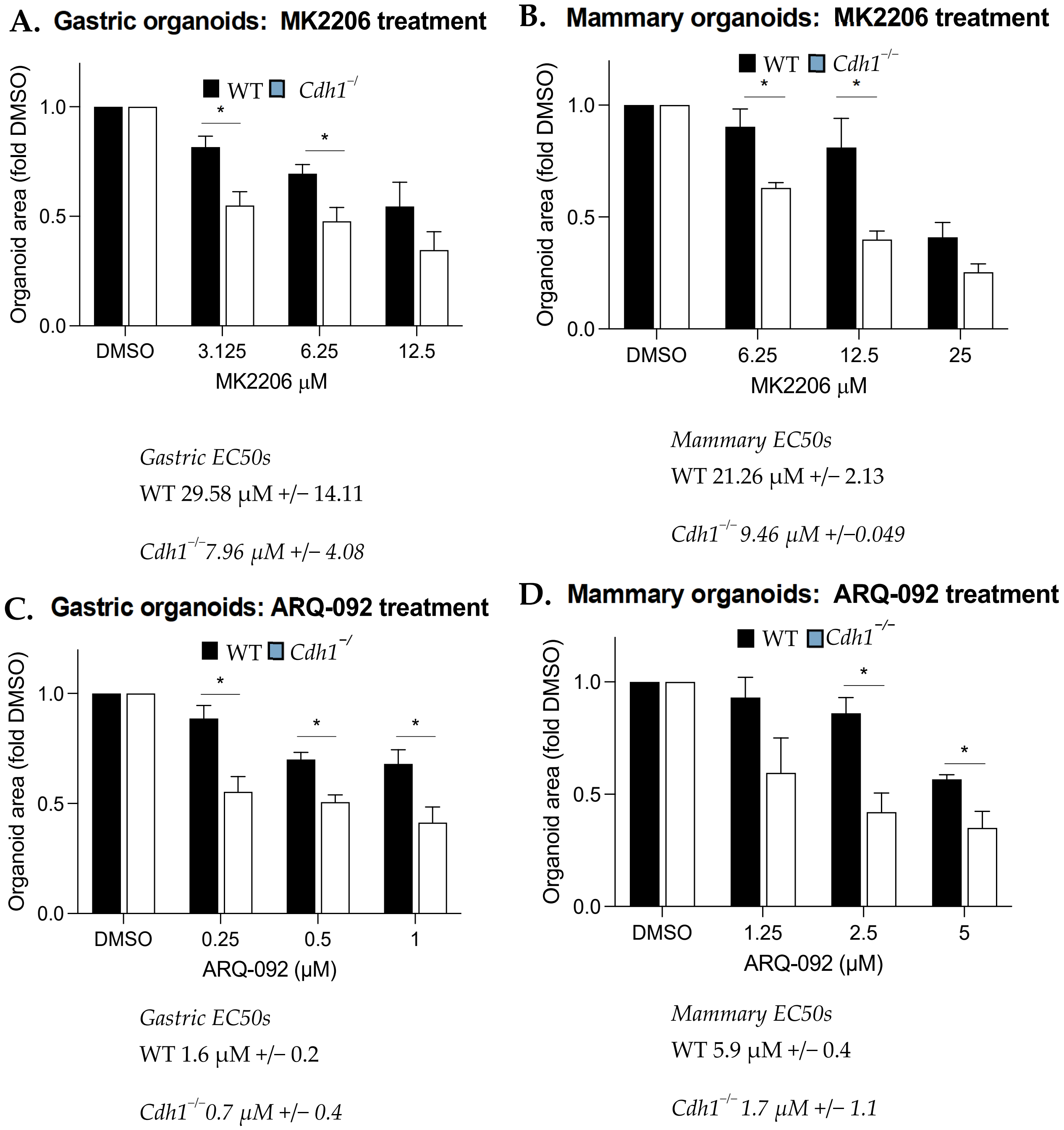

Figure S3. Mouse derived organoids containing *Cdh1*$^{-/-}$ cells are more sensitive to the growth inhibiting effects of allosteric AKT inhibitors. Bar graphs showing relative area of DMSO or MK2206 treated gastric (**A**) or mammary (**B**) organoids. Bar graphs showing relative area of DMSO or ARQ-092 treated gastric (**C**) or mammary (**D**) organoids. (For all graphs, error bars = SEM; * $p < 0.05$; $n \geq 3$ independent biological replicates; unpaired two-sided *t*-test.).

A.

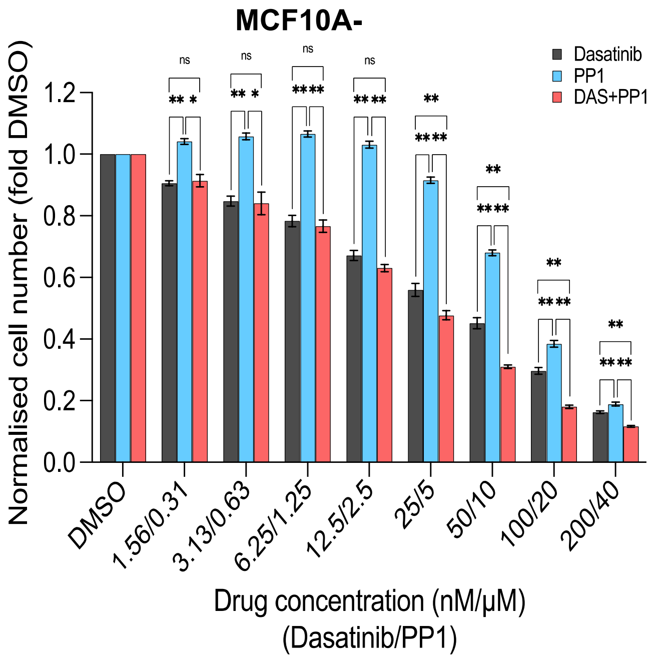
MCF10A-
Dasatinib
PP1
DAS+PP1
Normalised cell number (fold DMSO)
1.2
1.0
0.8
0.6
0.4
0.2
0.0
DMSO
1.56/0.31
3.13/0.63
6.25/1.25
12.5/2.5
25/5
50/10
100/20
200/40
Drug concentration (nM/µM)
(Dasatinib/PP1)

B.

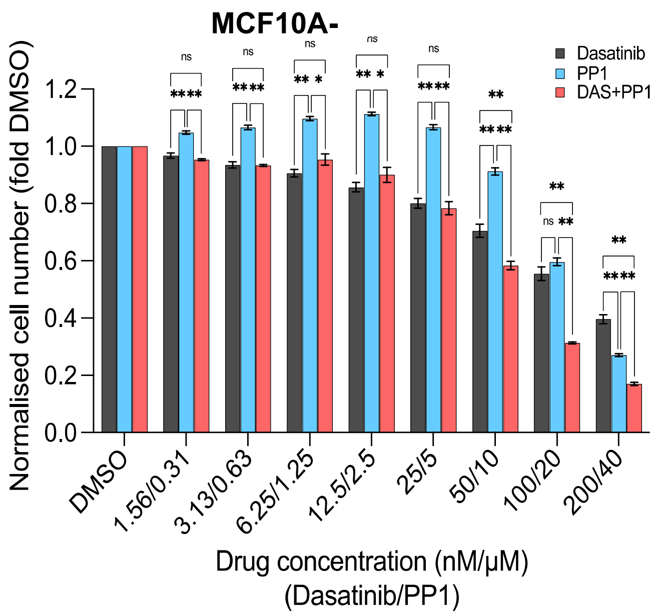
MCF10A-
Dasatinib
PP1
DAS+PP1
Normalised cell number (fold DMSO)
1.2
1.0
0.8
0.6
0.4
0.2
0.0
DMSO
1.56/0.31
3.13/0.63
6.25/1.25
12.5/2.5
25/5
50/10
100/20
200/40
Drug concentration (nM/µM)
(Dasatinib/PP1)

C.

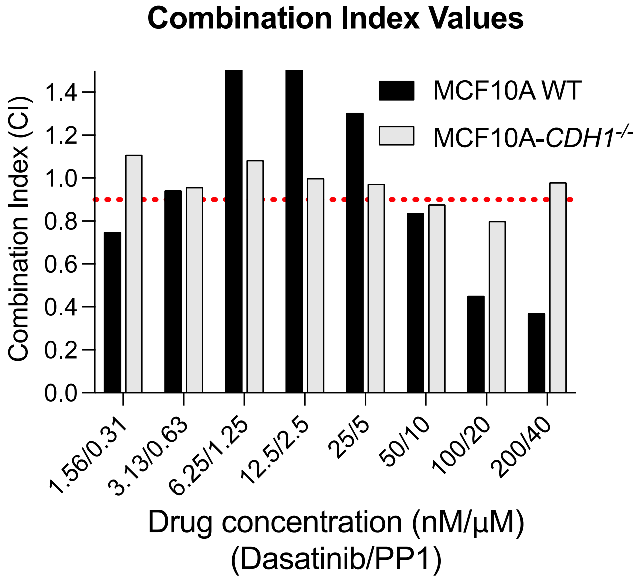
Combination Index Values
MCF10A WT
MCF10A-CDH1-/-
Combination Index (CI)
1.4
1.2
1.0
0.8
0.6
0.4
0.2
0.0
1.56/0.31
3.13/0.63
6.25/1.25
12.5/2.5
25/5
50/10
100/20
200/40
Drug concentration (nM/µM)
(Dasatinib/PP1)

Figure S4. Combining dasatinib with the SRC inhibitor PP1 is synergistic in MCF10A cells at higher concentrations. Normalised MCF10A-WT (**A**) and *CDH1*$^{-/-}$ (**B**) cell counts 48 h after treatment with serial dilutions of dasatinib, PP1 or a combination of dasatinib and PP1. X axis label, dasatinib concentration (nM)/PP1 concentration (μM). (**C**) Combination index (CI) values for MCF10a-WT and *CDH1*$^{-/-}$ cells treated with combination of dasatinib and PP1. Values below 0.9 indicate the drug combination is synergistic at that concentration. (For all graphs, error bars = SEM; ns: $p > 0.05$, * $p < 0.05$, ** $p < 0.01$; $n \geq 3$ independent biological replicates; unpaired two-sided t-test.).

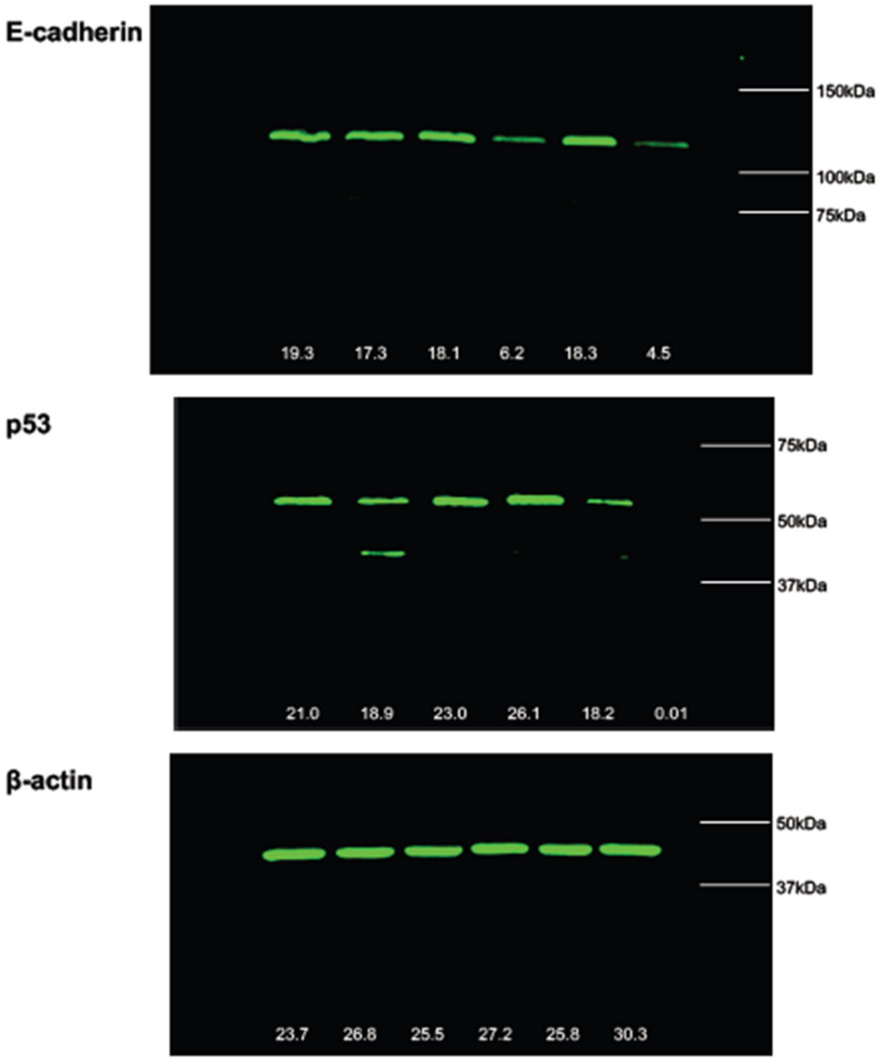

Figure S5. Uncropped western blots: mammary organoid induction test.

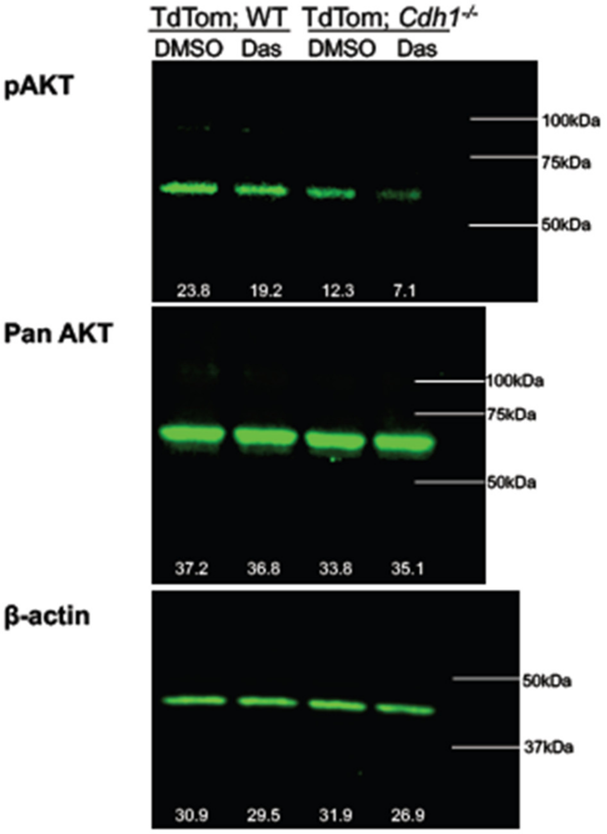

Figure S6. Uncropped western blots: gastric organoids +/− dasatinib.

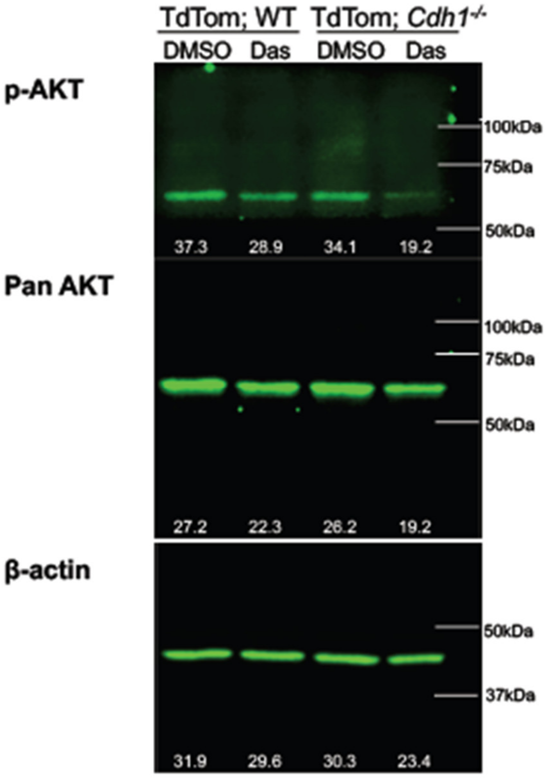

Figure S7. Uncropped western blots: mammary organoids +/− dasatinib.

Table S1. Organoid media components.

Reagent	Mammary organoid media final concentration	Gastric organoid media final concentration	Reagent ordering information
Advanced DMEM/F12	N/A	N/A	Life Technologies, #1864109
Pen/Strep	1x	1x	Life Technologies, #15140148
Glutamax	2 mM	2 mM	Life Technologies, #35050061
Hepes	10 mM	10 mM	Thermo Fisher, #15630080
B27	1x	1x	Thermo Fisher, #17504044
N-Acetyl-L-cysteine	1.25	1 mM	Sigma Aldrich, #A9165
EGF	50 ng/mL	50 ng/mL	Sigma Aldrich, #E9644
FGF10	10 ng/mL	100 ng/mL	Life Technologies, #PHG0204
A-83-01	1 uM	2 uM	Cayman Chemical, #9001799
Y-27632 dihydrochloride	5 uM	10 uM	Sigma Aldrich, #Y0503
Wnt3a conditioned media	2.50%	50%	From Hub-Wnt3A cells
R-spondin conditioned media	0.50%	10%	From 293T-HA-Rspo1-Fc cells
Noggin conditoned media		10%	From Hek293-mNoggin-Fc cells
N2		1x	Thermo Fisher, #17502048
Gastrin I		10 nM	Sigma Aldrich, #G9145
Insulin	5 ug/mL		Sigma Aldrich, #I0516
Hydrocortisone	100 ng/mL		Sigma Aldrich, #H0888
FGF2	5 ng/mL		Peprotech, #450-33

Table S2. Genes that were associated with both *AKT1* and *AKT3*. These were removed from the analysis before drug target selection to avoid selecting compounds that target both *CDH1*+ and *CDH1*-gastric cancers (as there is no correlation between *AKT1* and *CDH1* status.

Genes Associated with AKT3 and AKT1
FBN1
A2M
COL14A1
FERMT2
ANTXR1
TMEM47
ZEB1
RAB31
FRMD6
SPARCL1
DCN
GNB4
CYBRD1
RSPO3
AKAP12
CYP1B1
PRRX1
VCAN
MGP
EFEMP1
OGN
CFH
PHLDB2

ABI3BP
SPON1
CTSK
CXCL12
C7
GPNMB
FGL2
ABCA8
PI15
CPA3

Table S3. The 51 significantly *AKT3* associated genes with a fold change in expression >2 between high and low *AKT3* expressing samples in both the GEO and TCGA datasets. Fold change in expression and Benjamini-Hochberg-adjusted p-value in each dataset are shown for each gene. Genes are sorted based on the average of ranks assigned to the Benjamini-Hochberg-adjusted *p*-values in each dataset (not shown).

	TCGA FC	GEO FC	TCGA Adj *p*-Value	GEO Adj *p*-Value
DDR2	5.13	2.04	6.84E-51	2.89E-35
CDH11	3.14	2.33	1.99E-43	1.26E-31
AOC3	4.65	2.45	2.91E-43	1.57E-30
MSRB3	4.12	2.06	9.74E-43	6.82E-30
FSTL1	2.61	2.07	9.96E-43	4.76E-29
DPYSL3	4.28	2.07	4.95E-42	1.46E-28
COL8A1	5.33	2.02	8.41E-42	9.60E-28
TIMP3	3.16	2.16	9.55E-40	1.96E-27
CCDC80	5.29	2.28	9.89E-39	5.48E-27
LTBP1	3.00	2.10	1.54E-38	4.74E-26
PDLIM3	4.40	2.50	2.10E-38	1.85E-25
C1S	2.87	2.37	3.61E-36	2.66E-25
GREM1	6.27	2.35	9.83E-36	3.64E-25
GLT8D2	2.78	2.18	2.28E-35	1.88E-24
GAS1	5.25	3.11	2.37E-35	2.77E-24
TNS1	3.67	2.35	5.32E-35	5.75E-24
KCNMA1	7.31	3.77	3.20E-34	1.16E-23
MYLK	4.59	2.61	3.98E-34	1.31E-23
C1R	2.76	2.14	6.15E-34	2.33E-23
ADAMTS1	2.75	2.08	1.82E-33	3.75E-23
COL6A3	2.67	2.01	1.19E-32	4.89E-23
PLN	6.78	2.05	1.39E-32	1.01E-22
CALD1	3.09	2.71	1.49E-32	1.55E-22
FNDC1	6.00	2.79	4.85E-32	1.81E-22
IGFBP5	3.01	2.49	7.49E-32	6.52E-22
VIM	2.08	2.30	1.68E-31	2.83E-21
FHL1	4.38	2.30	7.42E-31	5.52E-21
SYNPO2	7.38	2.65	9.73E-31	5.91E-21
ACTA2	3.36	2.10	1.43E-30	9.50E-21
ASPN	3.96	2.49	2.41E-30	1.82E-20
SERPINF1	2.92	2.45	3.15E-30	4.59E-20
TAGLN	4.09	2.63	8.38E-29	1.34E-18
COL15A1	2.38	2.06	3.37E-28	3.06E-18
SDC2	2.13	2.54	4.76E-28	1.21E-17
SULF1	3.36	2.13	4.96E-28	2.28E-17
MMP2	2.70	3.14	1.85E-27	4.33E-17
MYH11	8.70	2.28	2.89E-27	4.98E-17
TNC	3.57	2.48	8.26E-27	6.60E-17
FN1	3.20	2.28	2.73E-26	1.23E-16
SFRP4	7.87	2.49	3.86E-26	7.99E-16
THBS2	3.68	2.07	9.61E-26	1.63E-15

CNN1	6.75	2.15	2.87E-25	1.18E-14
SFRP2	9.10	2.04	6.17E-25	2.56E-14
CAV1	2.46	2.24	2.18E-23	6.84E-13
SYNM	5.74	2.69	1.51E-22	1.08E-12
FBLN1	3.69	2.12	2.27E-22	1.18E-12
ACTG2	6.18	2.67	2.54E-20	2.17E-12
POSTN	2.33	2.17	1.95E-16	3.90E-12
NNMT	2.16	2.43	6.04E-16	1.79E-11
CTHRC1	2.27	2.12	8.32E-15	7.70E-10
CCL11	2.21	2.08	1.53E-08	4.04E-08
